# Synergistic Effect of Binary Mixed-Pluronic Systems on Temperature Dependent Self-assembly Process and Drug Solubility

**DOI:** 10.3390/polym10010105

**Published:** 2018-01-22

**Authors:** Chin-Fen Lee, Hsueh-Wen Tseng, Pratap Bahadur, Li-Jen Chen

**Affiliations:** 1 Department of Chemical Engineering, National Taiwan University, Taipei 10617, Taiwan; nattianan24@gmail.com (C.-F.L.); r00524034@ntu.edu.tw (H.-W.T.); 2 Department of Chemistry, Veer Narmad South Gujarat University, Surat 395007, India; pbahadur2002@yahoo.com

**Keywords:** polymer micelles, Pluronic, mixed micelle, micellization, synergistic effect, differential scanning calorimetry

## Abstract

Mixed Pluronic micelles from very hydrophobic and very hydrophilic copolymers were selected to scrutinize the synergistic effect on the self-assembly process as well as the solubilization capacity of ibuprofen. The tendency of mixing behavior between parent copolymers was systematically examined from two perspectives: different block chain lengths at same hydrophilicity (L92 + F108, +F98, +F88, and +F68), as well as various hydrophobicities at the same PPO moiety (L92 + F88, +F87, and +P84). Temperature-dependent micellization in these binary systems was clearly inspected by the combined use of high sensitivity differential scanning calorimeter (HSDSC) and dynamic light scattering (DLS). Changes in heat capacity and size of aggregates at different temperatures during the whole micellization process were simultaneously observed and examined. While distinction of block chain length between parent copolymers increases, the monodispersity of the binary Pluronic systems decreases. However, parent copolymers with distinct PPO moieties do not affirmatively lead to non-cooperative binding, such as the L92 + P84 system. The addition of ibuprofen promotes micellization as well as stabilizes aggregates in the solution. The partial replacement of the hydrophilic Pluronic by a more hydrophobic Pluronic L92 would increase the total hydrophobicity of mixed Pluronics used in the system to substantially enhance the solubility of ibuprofen. The solubility of ibuprofen in the 0.5 wt % L92 + 0.368 wt % P84 system is as high as 4.29 mg/mL, which is 1.4 times more than that of the 0.868 wt % P84 system and 147 times more than that in pure water at 37 °C.

## 1. Introduction

Block copolymer molecules undergo self-assembly in solvent, which has preferential solubility for one of the blocks. With a progressive increase in concentration, polymer micelles transform to hexagonal, lamellar structures and further spontaneously pack into crystal lattices. In some amphiphilic block copolymer aqueous solutions, temperature plays a crucial role in solvent selectivity of certain polymer blocks. Micelles exhibit the property of thermosreversibility between unimers and organized aggregates. For example, Pluronics (or Poloxamers), a kind of amphiphilic block copolymers of poly (ethylene oxide)_n_-poly(propylene oxide)_m_-poly(ethylene oxide)_n_, are well known to form various kinds of aggregates and biocompatible properties.

Micellization arises from the entropy-driven process where the middle poly (propylene oxide) block gets dehydrated and shrinks to form core-shell aggregates while system temperature increases. Micelles formed by amphiphilic block copolymers would increase hydrophobic drug solubility, metabolic stability, and circulation time [1]. As a promising nanomedicine carrier for anti-cancer drugs, polymer micelles have been evaluated in several clinical trials [2,3]. Due to poor aqueous solubility and systematic toxicity, anticancer drugs are extremely limited in terms of clinical application. Most chemotherapeutic drugs have a narrow therapeutic window and short elimination half-life so that higher doses are needed. However, the toxicity in the formulation would limit the maximum intravenous dose that can be used safely. Hence, Pluronics have been more and more attractive as drug carriers due to their non-toxicity.

A wide array of Pluronics is accessible depending on their molecular characteristics through varying the propylene oxide (PO)/ethylene oxide (EO) composition ratio and/or its molecular weight. Certainly, thermophysical properties of Pluronics micelles and their applications to controlled drug release have been extensively examined [4,5]. It has been found that Pluronic block copolymers are able to interact with multi–drug resistance (MDR) cancer cells leading to drastic sensitization of these tumors with respect to doxorubicin and other anticancer agents [6]. Individual Pluronic micelles have been studied for the solubilization capacity of drugs in pharmaceutical applications [7,8,9,10].

Mixed Pluronic systems have also been investigated as well to search for better formulations. For example, Gaisford et al. [11] suggested that similar PPO moiety would perform cooperative binding between two copolymers, while a distinct length of PPO does not. Oh et al. [12] conducted a series of tests of system stability on several mixtures consisting of extremely hydrophobic and hydrophilic Pluronics. It was found that the combination of L121 + F127 in a 50/50 mixture of a similar PPO moiety is the most stable one and shows an outstanding capacity to solubilize hydrophobic dyes. Many studies were then conducted to study the practical use of binary Pluronic systems based on the perspective of two copolymers with a similar PPO moiety [1,8,13,14]. From those reports, it was evidenced that the combination of a hydrophobic copolymer and a hydrophilic copolymer with a similar PPO chain length could be an ideal candidate for drug delivery and pharmaceutical use. In order to overcome low drug loading efficiency and the MDR-caused failure of chemotherapy in the treatment of cancer, Chen et al. [8] chose the formulation of P105 + F127 for the high sensitizing MDR cancer efficacy of P105, as well as the relatively similar hydrophobic moieties of F127.

On the other hand, the distinct PPO chain length between parent copolymers performing unimodal behavior to form mixed micelles was also observed. The F127 + L61 mixed system was used as one of the formulations for doxorubicin and is under clinical trial phase escalation [2]. Note that the F127 and L61 did not have a similar PPO moiety. Two combinations, F127 + P105 and F127 + L64, were applied to study the influence of the non-identical PPO block chain on the mixing behavior [15]. It was pointed out that not only Pluronics with a similar PPO moiety show cooperative binding, but distinct PPO chain lengths could also exhibit unimodal behavior via cooperative binding.

Micelles with spherical conformation are usually stable in aqueous solutions because of their core-shell structure. On the other hand, the lamellar and cylindrical morphology provides stable nano-environments from the interstitials between copolymers on the continuous architectures. It was calculated by the molecular theory of solubilization [16] and then experimentally evidenced for highly capable drug loading, but it was usually large and unstable in an aqueous solution [12]. Herein, use of mixtures of Pluronic block copolymers is an alternative to compensate for drawbacks of a neat system. The stability of the more hydrophobic copolymer in an aqueous solution could be substantially improved by adding another hydrophilic one. In other words, the solubilization capacity of the hydrophilic copolymer for drugs could be enhanced by the presence of the more hydrophobic one. Hence, the main advantage of the binary mixed Pluronic systems is to overcome the limitations of each neat system, allowing copolymer rearrangement to form stable nano-environments to enhance drug solubility.

Intrigued by those interesting studies, we conducted a series of experiments to study mixing behavior from two different perspectives: the different block chain length at the same hydrophilicity (L92 + F108, +F98, +F88, and +F68), as well as various hydrophobicities at same PPO moiety (L92 + F88, +F87, and +P84). Pluronic L92 was chosen as the hydrophobic parent copolymer and the other constituent was selected based on the two perspectives to systematically vary the resemblance between parent copolymers. Temperature-dependent micellization in binary Pluronic systems was inspected by the combined use of the high-sensitivity, differential scanning calorimeter (HSDSC) and dynamic light scattering (DLS). Heat capacity changes and aggregate sizes at different temperatures during the whole micellization process were scrutinized and correlated. Moreover, we used a nonsteroidal anti- inflammatory drug ibuprofen into the binary systems to examine the size distribution and solubilization capacity. This study endeavored to clarify the tendencies between mixing behaviors and tried to find out some principles for establishing binary systems.

## 2. Materials and Methods

### 2.1. Materials

**Figure 1:**
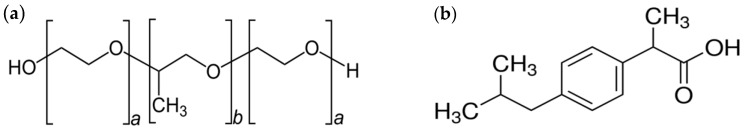
Molecular structures of (**a**) Pluronic and (**b**) Ibuprofen. Molecular weight of ibuprofen: 206.28 g/mol; solubility in water at 20 °C: 0.021 mg/mL [22]; partition coefficient (logP): 2.48 [23]; pKa: 5.38 at 25 °C [24].

Pluronics L92 and F108 were purchased from Sigma-Aldrich, and Pluronics F98, F88, F68, F87, and P84 were purchased from BASF Corporation. All these Pluronics were used as received without further purification. Their physicochemical and molecular characteristics are described in Table 1 and the molecular structure of Pluronic is schematically illustrated in Figure 1a. The drug α-methyl-4-(isobutyl) phenylacetic acid, known as ibuprofen, was bought from Alfa-Aesar and used as received. The molecular structure of ibuprofen is illustrated in Figure 1b. NaOH was bought from SHOWA, and its purity is 96%. Water was purified by double distillation followed by a PURELAB Maxima Series (ELGA Lab Water) purification system with a resistivity better than 18.2 MΩ cm.

### 2.2. High Sensitivity Differential Scanning Calorimetry (HSDSC)

HSDSC (VP-DSC, MicroCal) was used to determine the critical micelle temperature (CMT). After loading the reference and sample cells, the cell port air space was compressed to reach positive pressures up to approximately 0.2 MPa by using the pressurizing cap equipped with an o-ring-sealed piston. The detailed experimental procedure can be found in our previous studies [25,26,27]. All experiments were conducted at a scanning rate of 30 or 60 °C/h from 5 to 120 °C for 7 scans and reproduced in duplicate. The standard deviation of the CMT is always within ±0.15 °C for replicate measurements.

### 2.3. Dynamic Light Scattering (DLS)

Zetasizer Nano system equipped with a He-Ne laser operating at a wavelength 633 nm (Nano-ZS, Malvern, UK) was used to determine particle size distributions of Pluronic aggregates in solution. The system temperature can be controlled in a range of 0–80 °C. The intensity basis of the DLS data was adopted and presented in the study.

### 2.4. UV/Vis Spectroscopy and Drug Solubility

To prepare saturated drug-loaded solutions, an excess of drug (ibuprofen) powder was mixed with Pluronic aqueous solutions through stirring at 140 rpm at 37 °C for one day. Solutions were filtered by a syringe fitted with a 0.22 μm PTFE filter (Millipore) to remove unsolubilized drug before UV detection. The dissolved ibuprofen concentration in the solution was determined by measuring absorbance at 264.8 nm by using UV-Vis double beam spectrophotometer (CARY 100nc, Agilent Technologies, Santa Clara, CA, USA). Blank experiments without copolymer were conducted for the determination of drug solubility. Dilute solutions of ibuprofen dissolved in 0.1 N NaOH solution were used for the calibration according to the Beer-Lambert law, as illustrated in Figure S1 in the Supplementary Materials. The absorbance peak maximum of ibuprofen in neat and binary Pluronic solution was consistent with that in 0.1 N NaOH solution [28], as illustrated in Figure S2 in the Supplementary materials, which validates the applicability of this calibration curve. Some other details about this calibration curve can be found in the Supplementary Materials. For each neat and binary Pluronic system, at least 3 samples were prepared to perform the solubility measurements. Standard deviation (sd) of these multiple measurements for each system was determined by $\mathrm{sd}=\sqrt{\frac{\sum_{i=1}^{N}{\left(x_{i}-\bar{x}\right)}^{2}}{N-1}}$, where $\bar{x}$ stands for the average value of solubility.

## 3. Results and Discussion

In this study, six Pluronics, F108, F98, F88, F68, F87, and P84, in water, were chosen to examine their self-assembly process and drug (ibuprofen) solubility by using HSDSC, DLS, and UV-Vis spectroscopy. In addition, the synergistic effect of binary mixed Pluronic systems by adding L92 to each Pluronic mentioned above on the self-assembly process and drug (ibuprofen) solubility were also explored. Four Pluronics, F108, F98, F88, and F68, in water, were applied to systematically examine the effect of molecular weight of Pluronics with same hydrophobicity on the aggregation behavior of pure and mixed Pluronic L92 + Fx8 in water. On the other hand, three Pluronics F88, F87 and P84, were used to examine effect of hydrophobicity of Pluronics at a fixed PPO block length on the aggregation behavior of pure and mixed Pluronic L92 + F8x in water. In addition, the molecular weight and hydrophobicity of Pluronic copolymers were also manipulated to explore how to enhance the solubility of drug (ibuprofen).

### 3.1. Effect of Molecular Weight of Pluronic Fx8 (x = 6, 8, 9 and 10) at a Fixed PEO/PPO (80/20) Mass Ratio on the Thermophysical Properties of Pure and Mixed Pluronic L92 + Fx8 in Water

**Figure 2:**
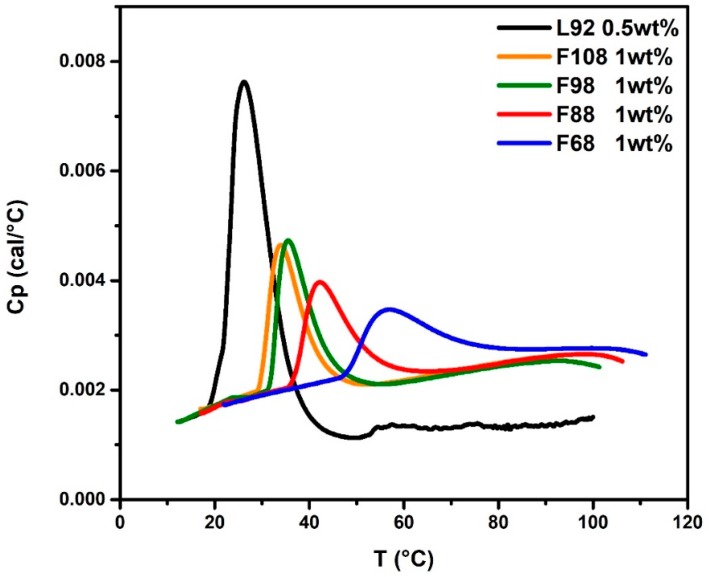
The HSDSC thermograms of pure Pluronic solutions.

First of all, the influence of molecular weight of Pluronic Fx8 on the self-assembly process of mixed Pluronic L92 + Fx8 mixtures was systematically examined. Four very hydrophilic Pluronics: F108, F98, F88, and F68, with a fixed PEO/PPO mass ratio (80/20), were chosen. The HSDSC thermograms of pure 1.0 wt % solutions of these Pluronics along with 0.5 wt % L92 in water are shown in Figure 2. Molar ratios of Fx8/L92 are 0.50, 0.56, 0.64, and 0.87 for *x* = 10, 9, 8 and 6, respectively. Usually, there are three methods applied to determine the CMT, i.e., the onset temperature *T*_onset_, the inflection point temperature *T*_inf_, and the peak maximum temperature *T*_m_ from the thermograms [29]. For example, the CMT (*T*_onset_) of F108, F98, F88, and F68 are 29.4, 30.7, 35.9, and 47.4 °C respectively. It is obvious that the CMT increases with decrease in the molecular weight for the Pluronic Fx8.

**Figure 3:**
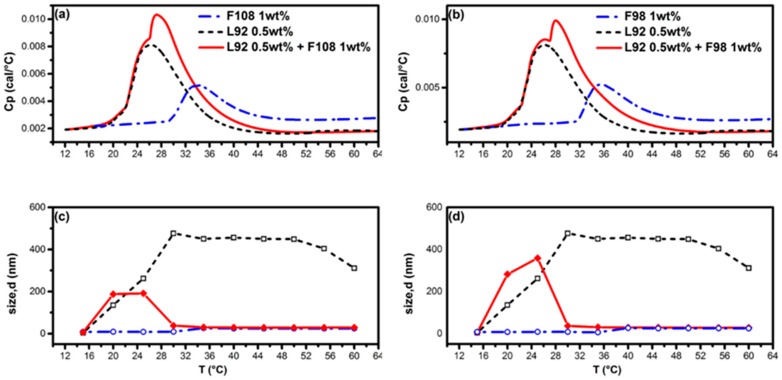
HSDSC thermograms (**a**,**b**) and evolution of size of aggregates (**c**,**d**) as a function of temperature from DLS; (**a**,**c**) Mixed Pluronic 0.5 wt % L92 + 1 wt % F108 system; (**b**,**d**) Mixed Pluronic 0.5 wt % L92 + 1 wt % F98 system. Red solid line and diamond (◆) for mixed Pluronic system. Black dashed line and open square (**□**) for L92. Blue dashed-dotted line and open circle (**○**) for F108/F98.

For 0.5 wt % solution of L92 system, phase separation occurred around 25 °C to form large aggregates of size of 450 nm, due to its hydrophobic nature as illustrated by DLS results in Figure 3c. The variation of aggregate size as a function of temperature for all systems is listed and reported in Tables S1–S6 in the Supplementary Materials. Nagarajan [16] pointed out that Pluronic L92 aggregates are lamellar structures because of their short PEO chain. The apparent hydrodynamic diameter of micelles formed by pure 1.0 wt % F108 is approximately 23.8 nm. The mixed Pluronic 0.5 wt % L92 + 1.0 wt % F108 system still exhibits large aggregates of ~190 nm in size below 25 °C, comparable to the size of aggregates formed by neat L92. Further increase in temperature up to 30 °C would dramatically decrease the micelle size to around 30.3 nm. It is interesting to note that the HSDSC thermogram illustrated in Figure 3a demonstrates a second peak with *T*_m_ = 27.2 °C appearing right after the 1st endothermic peak with *T*_onset_ = 19.0 °C (initiation of phase separation due to L92). This second peak is located between the endothermic peaks of neat L92 (*T*_onset_ = 18.9 °C) and F108 (*T*_onset_ = 29.4 °C). The appearance of this second peak suggests that F108 actively participates in the micellization process at *T*_m_ = 27.2 °C by integrating the L92 into mixed F108/L92 micelles via breaking down the large aggregates of L92, as revealed by sudden drop in micelle size around 30 °C illustrated in Figure 3c. The size of mixed F108/L92 micelles remains almost constant ~30 nm within the temperature range from 30 to 60 °C. The DLS results of the L92 + F108 system show a single sharp peak with a narrow distribution (polydispersity index (PDI) < 0.2) [30]: PDI = 0.17 at 25 °C and PDI = 0.10 at 35 °C. Note that the PPO block length of F108 (with 48 PO units) is almost equal to that of L92 (with 50 PO units). The similar PPO moiety between F108 and L92 indeed enhances the cooperative binding between these two copolymers to form mixed micelles, consistent with the findings of Oh et al. [12].

Instead of F108, a slightly shorter PPO block length of F98 (with 45 PO units) was applied to examine the self-assembly process of the mixed Pluronic 0.5 wt % L92 + 1.0 wt % F98 system. Figure 3b illustrates the HSDSC thermogram of the mixed Pluronic L92 + F98 system. The micellization process of the mixed Pluronic L92 + F98 system is rather similar to that of the L92 + F108 system. A second peak appears at 28.0 °C (T_m_), which is also between the endothermic peaks of neat L92 (*T*_onset_ = 19.0 °C) and F98 (*T*_onset_ = 30.7 °C). The diameter of micelle formed by pure F98 is approximately 25.0 nm, as the DLS data shown in Figure 3d. Addition of F98 to the L92 system breaks down the size of L92 aggregates from 358.4 nm to 27.9 nm after the occurrence of the second peak (i.e., *T* > 30 °C). The size of mixed F98/L92 micelles remains almost constant ~28 nm within the temperature ranging from 30 to 60 °C. However, PDI value of the solution is 0.70 at 27 °C and remains around 0.55 at high temperatures. This implies that the F98 is not as capable as F108 to stabilize the mixed micelles with a narrow size distribution due to a shorter PPO block length of F98.

In these two binary mixed Pluronic L92 + F108 and L92 + F98 systems, the size of mixed micelles is slightly larger than the one formed by neat F108 and F98 systems, respectively, and pronouncedly much smaller than L92 aggregates. Furthermore, the size of mixed micelles is stable and remains almost constant even at high temperatures without further agglomeration.

**Figure 4:**
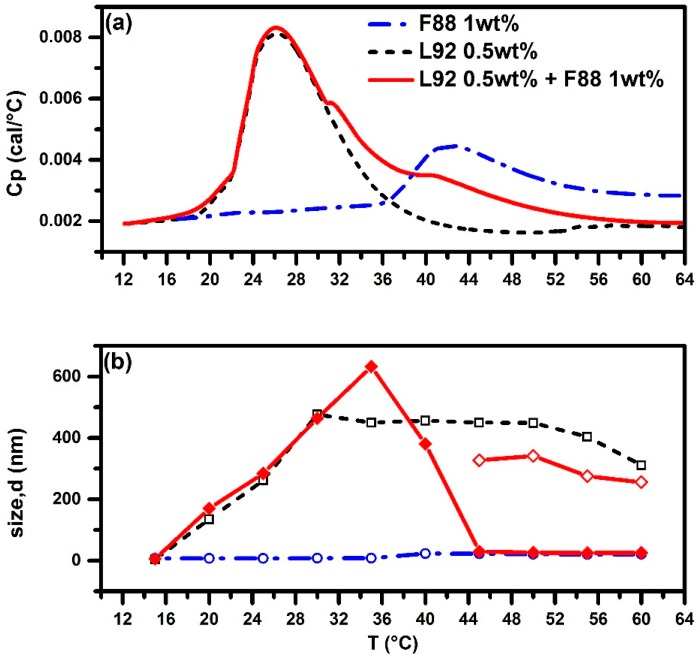
HSDSC thermograms (**a**) and evolution of size of aggregates (**b**) as a function of temperature from DLS for pure 0.5 wt % L92, pure 1 wt % F88, and mixed Pluronic 0.5 wt % L92 + 1 wt % F88 systems. Red solid line and diamond (◆) for L92 + F88. Red solid line and open diamond (**◇**) for L92 + F88 (the other coexisting aggregates). Black dashed line and open square (**□**) for pure L92. Blue dashed-dotted line and open circle (**○**) for pure F88.

Pluronic F88 with a shorter PPO block length (39 PO units) than that of F98 was then used to examine its capability of breaking down the L92 aggregates to form mixed micelles. It is interesting to find out that there appear two additional kinks at 31.2 °C (the second peak maximum temperature *T*_m2_) and 40.1 °C (the third peak maximum temperature *T*_m3_) in the HSDSC thermogram of the mixed Pluronic 0.5 wt % L92 + 1.0 wt % F88 system, as illustrated in Figure 4a. The DSC thermogram of the L92 + F88 system almost coincides with that of neat L92 system from 10 to 30 °C, as well as the temperature-dependent size of aggregates, as the DLS results shown in Figure 4b. This implies the presence of aggregates in the L92 + F88 system at temperatures below 30 °C are mainly formed by pure L92. Note that the second peak with *T*_m_ = 31.2 °C is located in-between the endothermic peaks of neat L92 (*T*_onset_ = 18.9 °C) and neat F88 (*T*_onset_ = 35.9 °C). Around this first kink at 31.2 °C in the DSC thermogram, the L92 + F88 system starts to deviate from that of neat L92 system. It is plausible to conjecture that F88 also participates in the aggregates to increase the size of aggregates up to 632 nm, which is larger than the size of aggregates formed by neat L92 system.

While the third peak (the second kink) with *T*_m3_ = 40.1 °C is located in-between the *T*_onset_ = 35.9 °C and *T*_m_ = 42.2 °C of the neat F88 system. When the temperature is increased up to 40.1 °C and beyond, F88 is able to break down the L92 aggregates of into smaller ones. With a further increase in temperature up to 44 °C and beyond, the L92 + F88 system exhibits bimodal behavior: small mixed F88/L92 micelles (~26.6 nm in diameter) and large aggregates (~300 nm in size) coexist. Note that the size of mixed L88/L92 micelles (~26.6 nm) is obviously larger than the size of micelles (~20.4 nm) formed by neat 1.0 wt % F88. The capability of F88 to break down the aggregates formed by L92 is not as efficient as for F108 and F98 as described before. Most of F88 molecules are incorporated into the mixed micelles (~26.6 nm) and only relatively small amount of F88 molecules join the aggregates formed by L92 (~300 nm).

**Figure 5:**
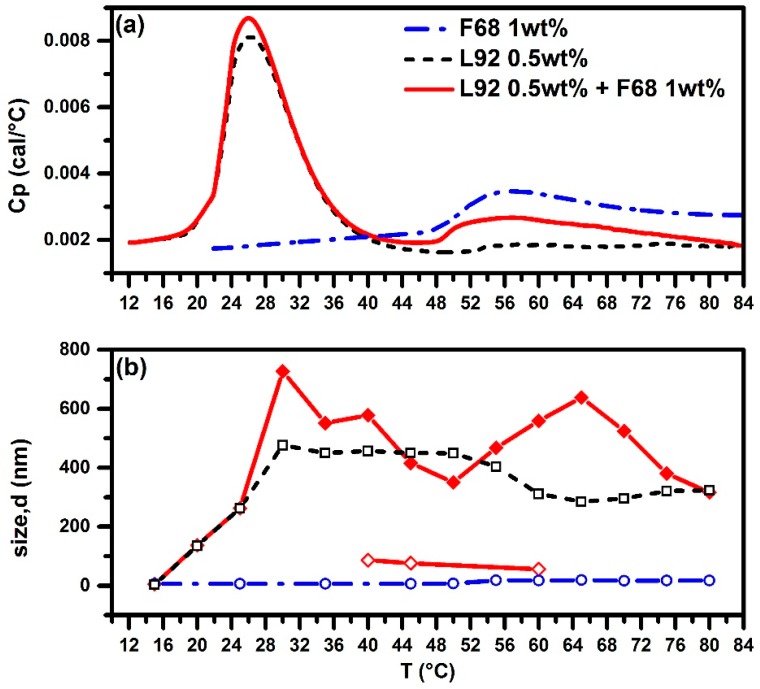
HSDSC thermograms (**a**) and evolution of size of aggregates (**b**) as a function of temperature from DLS for pure 0.5 wt % L92, pure 1 wt % F68, and mixed Pluronic 0.5 wt % L92 + 1 wt % F68 systems. Red solid line and diamond (◆) for L92 + F68. Red solid line and open diamond (**◇**) for L92 + F68 (the other coexisting aggregates). Black dashed line and open square (**□**) for pure L92. Blue dashed-dotted line and open circle (**○**) for pure F68.

There are only two peaks observed in the DSC thermogram of the 0.5 wt % L92 + 1.0 wt % F68 system, as shown in Figure 5a, compared to three peaks for the L92 + F88 system. Large aggregates (>400 nm) were observed at temperatures above 30 °C. Micelle size of neat F68 system is around 16.7 nm. It is obvious that the addition of F68 to L92 could not break down the size of large aggregates according to the DLS results shown in Figure 5b. It is interesting to note that the size of aggregates decreases to 350 nm at 45 °C while approaching the onset temperature of neat F68 system, but increases back to 467 nm at 55 °C. It may be attributed to the micellization of F68 by partially integrating in L92 aggregates. Very few small aggregates (~55 nm), less than 0.1% of the total number of aggregates, were observed in the temperature ranging from 40 to 60 °C. In other word, L92 + F68 system also exhibits bimodal behavior: small aggregates (~55 nm in size) and large aggregates (~400 nm in size) coexist within the temperature ranging from 40 to 60 °C.

**Figure 6:**
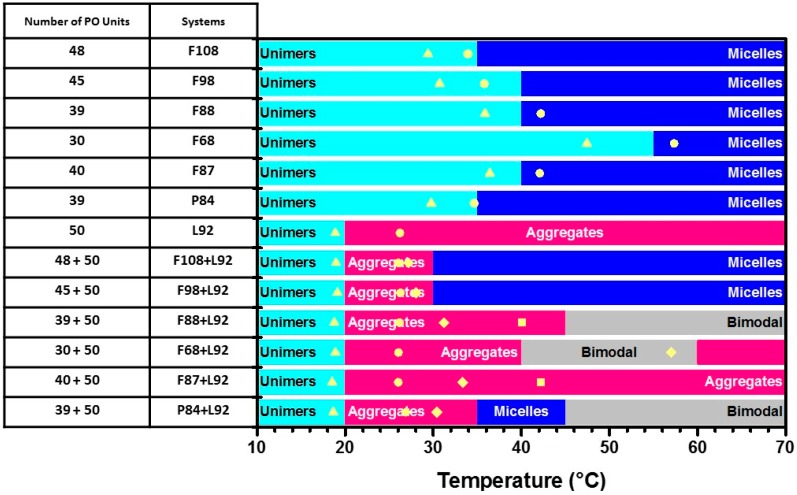
The evolution of self-assembly behavior of pure and mixed Pluronic L92 + Fx8 and L92 + F8x systems as a function of temperature. Symbols on the stacking bars represent characteristic temperatures measured by HSDSC. For pure Pluronic systems, *T*_onset_ (yellow triangle) and *T*_m_ (yellow circle) of the endothermic peak in the HSDSC thermograms. For the mixed Pluronic systems, *T*_onset_ (yellow triangle) of the first endothermic peak, T_m_ (yellow circle) of the 1st peak, *T*_m2_ (yellow diamond) of the 2nd peak, and *T*_m3_ (yellow square) of the 3rd peak in the HSDSC thermograms.

It is pronounced that the evolution of self-assembly process of mixed Pluronic L92 + Fx8 systems as a function of temperature depends on the molecular weight of copolymers. On initial heating of the mixed copolymer solutions, more hydrophobic L92 aggregates first form in the solution, and agglomeration may bring some hydrophilic Fx8 together into the aggregates. This could be evidenced by the larger aggregate sizes of mixed systems compared to the size of neat L92 system below 25 °C. With a further increase in temperature close to critical micelle temperature (CMT) of hydrophilic copolymer, dehydration of hydrophobic PPO block triggers the copolymers starting to aggregate. The performance of this second aggregation is then dominated by the block chain length of hydrophilic copolymer relative to the hydrophobic one. Large aggregates formed by neat L92 system could be suppressed by the introduction of hydrophilic Pluronic F108 with a large molecular weight. At a fixed PEO/PPO mass ratio of 80/20, Pluronic Fx8 with long PPO block length would disintegrate large aggregates of L92 dramatically to be integrated into the mixed Fx8/L92 micelles. In addition, the longer the PPO block length of hydrophilic copolymer Fx8, the more powerful the ability of Fx8 of breaking down the size of large aggregates to form stable mixed micelles in aqueous solution. For hydrophilic copolymers with a shorter PPO block, mixed Pluronic systems exhibit the coexistence of large aggregates and mixed micelles. Figure 6 summarizes the evolution of self-assembly process of pure and mixed Pluronic L92 + Fx8 systems as a function of temperature.

### 3.2. Effect of PEO Block Length of F8x (x = 8, 7 and 4) at a Fixed PPO Block Length on the Thermophysical Properties of Pure and Mixed Pluronic L92 + F8x in Water

**Figure 7:**
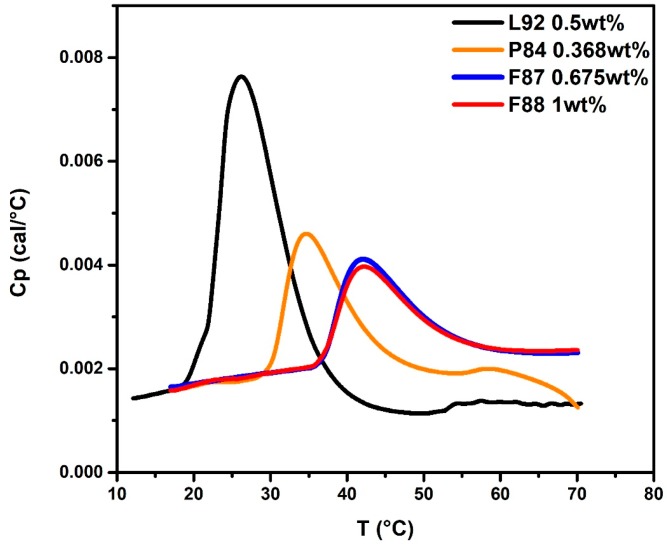
The HSDSC thermograms of pure Pluronic F88, F87, P84, and L92 system. 1.0 wt % F88, red line; 0.675 wt % F87, blue line; 0.368 wt % P84, orange line; and 0.5 wt % L92, black line.

The effect of PEO block length of Pluronic F8x (*x* = 8, 7, and 4) on the self-assembly process of mixed Pluronic L92 + F8x systems was systematically examined. Three Pluronics F88, F87, and P84 with a fixed PPO block length (~39 PO units) but varying PEO block length (respectively, 97, 61, and 19 EO units) were chosen in this study. In order to fix a constant amount of PO units of F8x added into the system, 1.0 wt % F88 was converted into 8.77 × 10^−7^ m F88 in water. Therefore, 8.77 × 10^−7^ m F87 and P84 aqueous solutions were equivalent to 0.675 wt % F87 and 0.368 wt % P84 in water. The HSDSC thermograms of pure 1.0 wt % F88, 0.675 wt % F87, 0.368 wt % P84, and 0.5 wt % L92 are compared and shown in Figure 7. Molar ratios of F8x/L92 are fixed at 0.64 for *x* = 8, 7 and 4. Note that the HSDSC thermogram of 0.675 wt % F87 system almost coincides with that of 1.0 wt % F88 system. It is obvious that CMT of 0.368 wt % P84 system is lower than that of the other two F8x systems. The CMTs (*T*_onset_) of the 1.0 wt % F88, 0.675 wt % F87, and 0.368 wt % P84 are 35.9, 36.4, and 29.8 °C, respectively.

**Figure 8:**
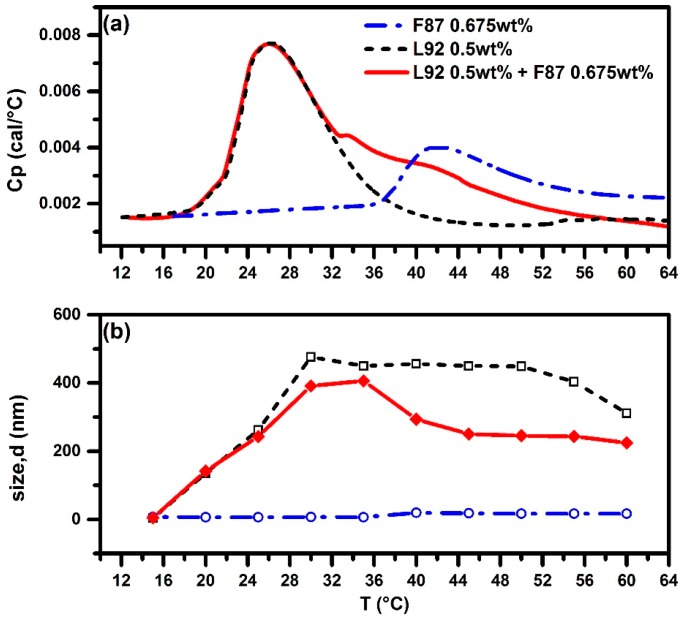
HSDSC thermograms (**a**) and evolution of size of aggregates (**b**) as a function of temperature from DLS for pure 0.5 wt % L92, pure 0.675 wt % F87, and mixed Pluronic 0.5 wt % L92 + 0.675 wt % F87 systems. Red solid line and diamond (◆) for L92 + F87. Black dashed line and open square (**□**) for pure L92. Blue dashed-dotted line and open circle (**○**) for pure F87.

There exist two kinks after the first endothermic peak in the HSDSC thermogram of the mixed Pluronic 0.5 wt % L92 + 0.675 wt % F87, as shown in Figure 8a, similar to that of 0.5 wt % L92 + 1 wt % F88 system (Figure 4a).

For the 0.5 wt % L92 + 0.675 wt % F87 system, the second and third peaks (two kinks) appear at *T*_m2_ = 33.3 °C and *T*_m3_ = 42.2 °C, respectively. However, evolution of size of aggregates as a function of temperature from the DLS results for the L92 + F87 system (Figure 8b) is rather different from that of the L92 + F88 system (Figure 4b). For the 0.5 wt % L92 + 1.0 wt % F88 system, major aggregates (in terms of number of aggregates) detected in the solution are mixed micelles of ~26 nm in size coexisting with very few large aggregates of ~300 nm in size when system temperature is higher than 45 °C. In contrast, for 0.5 wt % L92 + 0.675 wt % F87 system, only large aggregates around 245 nm are detected without any small aggregates when system temperature is higher than 40 °C. The PDI value after the second peak was around 0.37 and aggregate size distribution could be found in Figures S3 and S4 in the Supplementary Materials. Obviously, adding F87 to the L92 system does not break down the size of large aggregates like F88 does, since F87 is slightly more hydrophobic than F88 (the PEO block length of F87 is shorter than that of F88).

**Figure 9:**
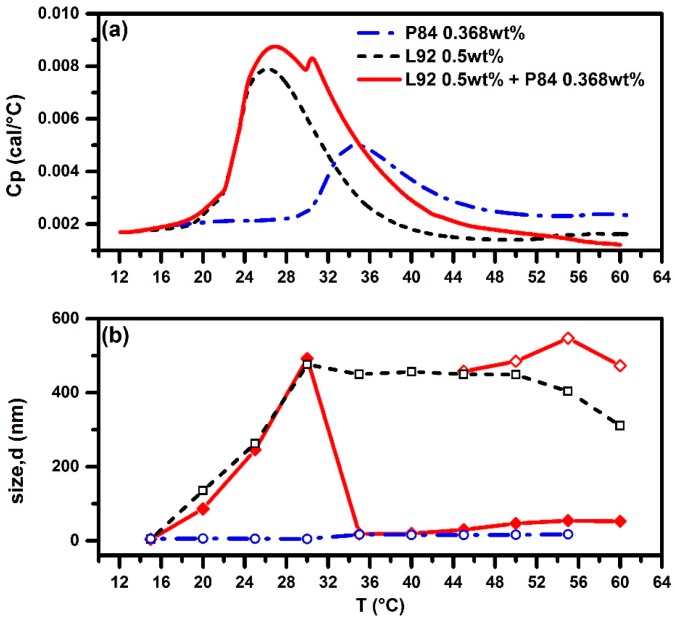
HSDSC thermograms (**a**) and evolution of size of aggregates (**b**) as a function of temperature from DLS for pure 0.5 wt % L92, pure 0.368 wt % P84, and mixed Pluronic 0.5 wt % L92 + 0.368 wt % P84 systems. Red solid line and diamond (◆) for L92 + P84. Red solid line and open diamond (**◇**) for L92 + P84 (the other coexisting aggregates). Black dashed line and open square (**□**) for pure L92. Blue dashed-dotted line and open circle (**○**) for pure P84.

Instead of F87, an even shorter PEO block length P84 (19 EO units) was used to prepare the mixed Pluronic 0.5 wt % L92 + 0.368 wt % P84 aqueous solution for comparison. The HSDSC thermogram of this L92 + P84 system, as illustrated in Figure 9a, shows that as the temperature is increased from 10 °C to 18.6 °C (=*T*_onset_), the first endothermic peak occurs to trigger phase separation due to the Pluronic L92 to form large aggregates. When the temperature is further increased up to ~30 °C, another endothermic peak appears for P84, precipitating the self-assembly process to form mixed P84/L92 micelles. Simultaneously, the size of aggregates dramatically drops from 492 nm down to about 18.3 nm, which is slightly larger than the size of micelles formed by neat P84 (16.5 nm), as revealed by DLS results shown in Figure 9b. PDI value is around 0.55, implying the polydispersity of size of aggregates in the solution, but the system is dominated by the mixed micelles (18.3 nm). When the temperature is further increased up to 45 °C and beyond, large aggregates about 450 nm are detected, but only in the proportion of less than 0.1% (on number basis), and coexisting with the mixed micelles. The observed large aggregates above 45 °C may arise from the intrinsic intention of phase separation for neat 1% P84 in water (cloud point = 74 °C). The hydrophobicity of these Pluronics increases along with temperature.

It is surprising that the addition of P84 would break down large aggregates and develop small aggregates (micelles) that stably exist in the solution. P84, more hydrophobic than F87 and F88, was predicted not to be as stable in the solution as F88 for the shorter PEO chain length, nor as being powerful enough to entangle with L92 molecules that break down the size of large aggregates as F108. At lower temperatures, Pluronic L92 stacks into a lamellar form. Increasing temperature to the CMT of 0.368 wt % P84 promotes molecular interaction between P84 and L92, owing to the driving force of aggregation for P84. This rather hydrophilic and slightly larger copolymer compared to L92 may be incorporated into lamellar structure mainly formed by L92 and gradually breaks down size of aggregate. Due to the dehydration of P84 molecules, micellization process of P84 would probably be accompanied by L92 molecules to form mixed micelles. However, owing to the shorter PPO block length of P84, the ability of entangling with L92 is limited and large aggregates still exist in the system. Raising temperature would enlarge the micelles and make them more hydrophobic.

For a binary-mixed Pluronic system including an extremely hydrophobic Pluronic copolymer (L92 used in this study) and a hydrophilic one, Pluronics with large molecular weight would disintegrate large aggregates pronouncedly, as well as diminish the cloud point at room temperature efficiently. Under the condition of a fixed PPO block length, Pluronic copolymers with a shorter PEO block length would, in contrast, disintegrate large aggregates more efficiently. The evolution of aggregation behavior of pure and mixed Pluronic L92 + F8x systems as a function of temperature is also summarized and illustrated in Figure 6.

### 3.3. Enhancement of Solubility of Ibuprofen in Neat and Mixed Pluronic Systems

The drug (ibuprofen) solubility in neat and mixed Pluronic systems was then carefully measured to examine the effect of molecular weight and hydrophilicity of copolymers. Firstly, solubility of ibuprofen in 1.5 wt % neat Pluronic F108, F98, F88, and F68 system at 37 °C was examined. It is obvious that solubilization capacity of F68 for ibuprofen is inferior to the other three Pluronics, and no significant differences exist among neat F108, F98, and F88 systems. However, based on the experimental results listed in Table 2, the capability of drug incorporation for the four copolymers could still be distinguishable. It is interesting to find out that the solubilization capacity of neat Pluronics with different block chain lengths at the same hydrophilicity is in the order of F98 > F108> F88 > F68. It seems that the micelles formed by longer block chain solubilize more ibuprofen, except F108. This may be attributed to the long PEO block length of F108 inducing a steric hindrance for the entrapment efficiency. Liveri et al. [31] performed a systematic spectrophotometric study of the kinetic of solubilization process of the poorly water soluble drug tamoxifen. They pointed out that PEO corona may act as the steric barrier that hampers the transfer of tamoxifen into the micelle core. Pluronic F108 has 127 EO units on each side of the copolymer, which may contribute a stable environment for the hydrophobic core inside the micelle with PEO block chains stretching toward the solution, but simultaneously may probably hinder the transfer of ibuprofen leading to limited incorporating amount of drugs.

Particle size distribution and PDI values for the systems with/without ibuprofen at 37 °C were also measured and used as an index to evaluate system stability. The micelle hydrodynamic diameters of neat Pluronic F108 and F98 system are 27.5 nm and 25.5 nm, respectively, whereas for ibuprofen-loaded micelles they are 26.6 nm and 24.6 nm, respectively. There is no obvious change in particle size after drug incorporation. It should be noted that the CMTs of neat (1.5 wt %) F88 and F68 system are higher than 37 °C. Therefore, the F88 and F68 molecules exist as unimers at 37 °C in neat F88 and F68 system without ibuprofen, as the particle size reported in Table 2. The addition of ibuprofen would trigger the micellization process at lower temperature 37 °C to form uniform micelles with around 25 nm in diameter, larger than the micelle size of the neat Pluronic system without ibuprofen (say at 60 °C, see Tables S3 and S4 in the Supplementary Materials). All of the PDI values drop down below 0.15 for these systems after adding ibuprofen.

In addition to the 1.5 wt % F88 system, solubility of ibuprofen in 1.175 wt % F87 and 0.868 wt % P84 system at 37 °C was carefully measured. Solubilization capacity of neat Pluronic F8x at a fixed PPO block length with various hydrophilicity is in the order of F88 ≅ F87 < P84, as reported in Table 3. It is pronounced that the solubility of ibuprofen increases along with an increase in the hydrophobicity of Pluronic used. Similar to the 1.5 wt % F88 system, the CMT of neat 1.175 wt % F87 system is also higher than 37 °C. Pluronic F87 molecules exist as unimers at 37 °C in neat F87 system without ibuprofen. Addition of ibuprofen to the Pluronic F87 system promotes the micellization at 37 °C to form uniform micelles of ~20 nm in diameter, larger than the micelle size of pure F87 system without ibuprofen, e.g., the micelle size of 0.675 wt % F87 system at 45 °C is ~18 nm. On the other hand, neat Pluronic F84 system already forms micelles of ~18 nm in diameter at 37 °C, and addition of ibuprofen to neat P84 system would increase the size of aggregates up to 88 nm to enhance the solubility of ibuprofen as high as 3.02 mg/mL, larger than that in the 1.5 wt % F108 system (1.61 mg/mL). This finding is consistent with that of Singla et al. [32]. They explored the solubilization of hydrophobic drug (oxcarbazepine) in different Pluronics F108, F127, and P84, revealing that the solubilization capacity of P84 is the highest among these three copolymers.

Note that the solubility of ibuprofen in pure water is as low as 0.0206 mg/mL at 35 °C and 0.0264 mg/mL at 40 °C [33], which can be linearly interpolated to estimate the solubility of ibuprofen at 37 °C around 0.0229 mg/mL. The solubility of ibuprofen in 1.5 wt % F98 system (1.75 ± 0.03 mg/mL) is dramatically increased by 75 times more than that in pure water. Furthermore, the solubility of ibuprofen in 0.886 wt % P84 is even increased up to 130 times more than that in pure water. All these Pluronics demonstrate the outstanding solubilization capacity for incorporating hydrophobic drug (ibuprofen).

Based on our experimental results, Pluronics with larger molecular weight (F98 in Fx8 series) and *more hydrophobic* characteristics (F84 in F8x series) exhibit better solubilization capacity for ibuprofen. To follow along this line, it is plausible to conjecture that the solubility of ibuprofen can be enhanced by simply increasing the hydrophobicity of Pluronic through replacing partially the Pluronic (0.5 wt % Fx8 or F8x) by a more hydrophobic Pluronic (0.5 wt % F92). Thus, all the 1.5 wt % Fx8 systems are replaced by the 1.0 wt % Fx8 + 0.5 wt % F92 systems. On the other hand, the 1.175 wt % F87 (and 0.868 wt % P84) system is replaced by the 0.675 wt % F87 + 0.5 wt % F92 (and 0.368 wt % P84 + 0.5 wt % F92) system for further exploration of enhancement of ibuprofen solubility.

For the binary mixed Pluronic L92 + Fx8 systems (see Table 2), L92 can be stabilized by F108 (and F98) to form mixed F108/L92 (and F98/L92) micelles of ~30 nm in diameter via cooperative binding between parent copolymers. Addition of ibuprofen dramatically increases the size of aggregates from 30 to 320 nm, forming a stable and rather monodisperse system where the PDI value is around 0.20 for these two L92 + F108 and L92 + F98 systems. For the L92 + F88 system, the size of aggregates drops down from 632 to 254 nm at 37 °C after ibuprofen is loaded. The size of aggregates was stabilized to around 30 nm after 45 °C for the L92 + F88 system without ibuprofen, as mentioned above. Below 45 °C, large aggregates (above 600 nm) with broad distribution were still observed in the solution. Inclusion of ibuprofen makes the size of aggregates decrease and become more uniform, and the PDI value dropped from 1.00 to 0.19. That is, the large aggregates (632 nm) in the solution could be stabilized by ibuprofen developing monodisperse distribution (254 nm). Meznarich and Love [34] revealed that methylparaben, a common food and drugs additive, enhances monodisperse micelles formed by Pluronic F127 and promotes its gelation behaviors on the kinetic studies. Similar trend was observed from the L92 + F68 system for the decrease in PDI value from 0.34 to 0.12. However, the size of aggregates after drug loaded does not change (from 550 to 590 nm), indicating that the block chain length of Fx8 is strongly associated with the capability to stabilize the aggregates. This observation again supports our conclusions: the longer the block chain length of the hydrophilic parent copolymer, the more capable this copolymer is to break down large aggregates. The addition of ibuprofen to the L92 + Fx8 system induces large aggregates in the solutions due to the insufficient amount of hydrophilic PEO chains that is necessary to assemble into micelles.

For all the mixed Pluronic L92 + Fx8 (*x* = 10, 9, 8, and 6) systems, the solubility of ibuprofen indeed is dramatically enhanced at least 2 times higher than that of neat Pluronic systems, as can be seen in the solubility data listed in Table 2. It is interesting to find out that the solubilization capacity of the mixed Pluronic L92 + Fx8 systems with different block chain lengths at the fixed PEO/PPO mass ratio (80/20) is in the order of F98 ≅ F108 ≅ F88 > F68. The solubility of ibuprofen in the mixed L92 + F98 system is 3.37 mg/mL, around 147 times larger than that in pure water.

For the mixed Pluronic L92 + F8x (*x* = 8, 7 and 4) systems, the addition of ibuprofen to the L92 + F87 and L92 + F88 systems would decrease the size of aggregates (see Table 3), as well as the PDI value compared to that without ibuprofen. Replacement of 0.5 wt % F87 by L92 in the system would enhance the solubility of ibuprofen by 1.8 times than that of neat F87 system. Self-assembly behavior of the mixed L92 + P84 system is similar to that of the mixed L92 + F108 system. The size of aggregates is increased from 18 to 130 nm before and after ibuprofen loaded, but the aggregates still remain rather monodisperse (PDI = 0.24) after ibuprofen loaded. Replacement of 0.5 wt % P84 by L92 would enhance the solubility of ibuprofen up to 4.29 mg/mL, around 147 times higher than that in pure water.

Lee et al. also demonstrated that solubilization capacity of pure P123 system was lower than that of the mixed Pluronic L121 + P123 system by using a water-insoluble dye Sudan Ш [1]. The lamella-forming L121 provides a hydrophobic pool to increase in the solubilization capacity compared to pure P123 system. Dutra et al. reported that solubilization capacity of P123 + F127 system for griseofulvin is higher than that of pure F127 and increases along with the proportion of P123 [35]. These observations [1,35] are in good agreement with our experimental results that the replacement of hydrophilic Pluronic by L92 in the systems increases solubility of ibuprofen compared to that in neat Pluronic systems. Indeed, either the mixed Pluronic L121 + P123 system or the P123 + F127 system is composed of parent copolymers with similar PPO moiety. It should be noted that the Pluronic L92 has 50 PO units and F68 has 30 PO units, as can be seen in the molecular structures of neat Pluronics illustrated in Table 1. That is, the difference of the numbers of PO units between L92 and F68 is as high as 20. The solubility of ibuprofen in the mixed 0.5 wt % L92 + 1.0 wt % F68 system is around 4 times larger than that in the 1.5 wt % F68 system. In other words, introducing more hydrophobic Pluronic L92 into the neat F68 system would obviously enhance the solubility of ibuprofen, even having quite different PPO block lengths between L92 and F68.

For all the six mixed Pluronic systems substituted by 0.5 wt % L92, the solubility of ibuprofen indeed is dramatically enhanced compared to that of neat Pluronic systems, as shown in Table 2 and Table 3. The partial replacement of the Pluronic (Fx8 or F8x) by a more hydrophobic Pluronic (F92) would increase the total hydrophobicity of Pluronics used in the system to enhance the solubility of ibuprofen.

## 4. Conclusions

Through the investigations of a series of binary mixtures concerning various block chain lengths and hydrophobicity, observations from the temperature-dependent self-assembly process using DLS and HSDSC demonstrated the tendencies between mixing behaviors. By simultaneously detecting heat capacity change and particle sizes, the evolution of PDI values reflect the mixing process along with temperature. The PDI value was around 0.50 at a lower temperature for the system L92 + F108, while it gradually decreased to 0.08, reflecting uniform mixed-micelles formation. On the other hand, the PDI value remained around 0.20 at a higher temperature for the system L92 + F68. For the binary Pluronic systems, while the distinction of block chain length between the parent copolymers increases, the monodispersity of the binary Pluronic systems decreases. However, the parent copolymers with distinct PPO moieties do not affirmatively lead to non-cooperative binding, such as L92 + P84. Small particle sizes around 30 nm were still detected after the CMT of P84 in the mixed system.

Ibuprofen was added into the mixed systems to observe solubilization capacity and stability. The addition of ibuprofen promotes micellization and stabilizes aggregates in the solution. Also, participation of the drug unifies the distribution of particle size developing a nearly monodisperse system. The solubility of ibuprofen in the mixed Pluronic systems is dramatically enhanced compared to that of the neat Pluronic systems for all the six mixed systems substituted by 0.5 wt % L92. The capability to incorporate ibuprofen into the system L92 + P84 is the most outstanding one: 4.29 mg/mL, which is 147 times more than that in pure water at 37 °C. In addition, the smallest particle size (130 nm) was measured from the L92 + P84 system.

From our systematic studies, the system of parent copolymers with a similar PPO moiety certainly leads to synergistic mixing (L92 + F108 or + F98), while the parent copolymer with intermediate hydrophobicity (P84) also has the ability to cooperatively bind with L92, forming well dispersing systems. With the participation of the model drug—ibuprofen—the solubilization capacity of the mixed Pluronic systems increases as the hydrophobicity of the system increases (L92 + F87 or + P84). It is interesting to point out that the particle size of the mixed systems decreases as the hydrophobicity of Pluronic increases. This may imply that the parent copolymers with a similar PPO moiety are suitable for drug encapsulation. Systems with distinct block chain lengths of parent copolymers, such as L92 + P84, demonstrate high solubilization capacity, as well as stability after drug incorporation. Additionally, the long-term stability of these binary Pluronic systems is crucial with regard to the practical applications of such systems. We are still in the process of examining the long-term stability of these binary Pluronic systems for further applications. Up until now, these binary Pluronic systems remain homogeneous at room temperature for at least one month. Our result also indicates that synergistic mixing of the systems with incorporated drugs could not only depend on the combinations of copolymers, but also the characteristics of drug-forming stable systems. Systematic studies from the series of experiments prove the influence of block chain length, as well as hydrophobicity, on the two copolymers.

## Figures and Tables

**Table 1:** Physicochemical characteristics of Pluronics used.

Pluronic	Average (Mw ^a^)	Structure [11,17,18,19,20,21]	HLB ^a^	CP at 1% ^a^ (°C)
L92	3650	(EO)_8_(PO)_50_(EO)_8_	1–7	26
F108	14,600	(EO)_127_(PO)_48_(EO)_127_	>24	>100
F98	13,000	(EO)_118_(PO)_45_(EO)_118_	28	>100
F88	11,400	(EO)_97_(PO)_39_(EO)_97_	28	>100
F68	8400	(EO)_80_(PO)_30_(EO)_80_	>24	>100
F87	7700	(EO)_61_(PO)_40_(EO)_61_	>24	>100
P84	4200	(EO)_19_(PO)_39_(EO)_19_	12–18	74

^a^ Information from BASF.

**Table 2:** Hydrodynamic Diameter of aggregates (*D*_h_, nm) and solubility of ibuprofen in neat Pluronic Fx8 (1.5 wt %) and binary mixed Pluronic 0.5 wt % L92 + 1.0 wt % Fx8 systems at 37 °C.

**Neat Pluronic**	**F108 (1.5 wt %)**	**F98 (1.5 wt %)**	**F88 (1.5 wt %)**	**F68 (1.5 wt %)**
*D_h_* (nm) without ibuprofen	27.5 ± 0.4	25.5 ± 0.4	6.8 ± 0.2	5.6 ± 0.2
*D_h_* (nm) Saturated ibuprofen	26.6 ± 0.2	24.6 ± 0.2	23.9 ± 0.1	25.2 ± 1.8
Solubility of ibuprofen (mg/mL)	1.35 ± 0.03	1.35 ± 0.08	1.18 ± 0.01	0.44 ± 0.11
**Mixed Pluronic**	**L92 (0.5 wt %)** **+ F108 (1 wt %)**	**L92 (0.5 wt %)** **+ F98 (1 wt %)**	**L92 (0.5 wt %)** **+ F88 (1 wt %)**	**L92 (0.5 wt %)** **+ F68 (1 wt %)**
*D_h_* (nm) * without ibuprofen	30.3 ± 0.1	30.3 ± 1.5	632 ± 25	550 ± 85
*D_h_* (nm) Saturated ibuprofen	327 ± 20	314 ± 11	254 ± 8	590 ± 27
Solubility of ibuprofen (mg/mL)	2.28 ± 0.04	2.41 ± 0.07	2.25 ± 0.11	1.75 ± 0.07

* The data measured at 35 °C.

**Table 3:** Hydrodynamic Diameter of aggregates (*D_h_*, nm) and solubility of ibuprofen in neat Pluronic F8x and binary mixed Pluronic L92 + F8x systems at the same total mass concentration at 37 °C.

**Neat Pluronic**	**F88 (1.5 wt %)**	**F87 (1.175 wt %)**	**P84 (0.868 wt %)**
*D_h_* (nm) without ibuprofen	6.8 ± 0.2	5.7 ± 0.1	17.9 ± 0.8
*D_h_* (nm) Saturated ibuprofen	23.9 ± 0.1	20.1 ± 0.1	88.2 ± 0.04
Solubility of ibuprofen (mg/mL)	1.18 ± 0.01	1.51 ± 0.04	2.74 ± 0.03
**Mixed Pluronic**	**L92 (0.5 wt %)** **+ F88 (1 wt %)**	**L92 (0.5 wt %)** **+ F87 (0.675 wt %)**	**L92 (0.5 wt %)** **+ P84 (0.368 wt %)**
*D_h_* (nm) * without ibuprofen	632 ± 25	406 ± 18	18.3 ± 1.0
*D_h_* (nm) Saturated ibuprofen	254 ± 8	144 ± 4	130 ± 7
Solubility of ibuprofen (mg/mL)	2.25 ± 0.11	2.28 ± 0.01	2.71 ± 0.24

* The data measured at 35 °C.

## Supplementary Materials

The following are available online at http://www.mdpi.com/2073-4360/10/1/105/s1, Figure S1. Calibration line of Ibuprofen in 0.1 N NaOH. Figure S2. UV absorbance of ibuprofen vs. wavelength in different solvents. Figure S3. Volume based Size Distribution of L92 + F87 system at 50 °C. Figure S4. Number based Size Distribution of L92 + F87 system at 50 °C. Table S1. Hydrodynamic Diameters of neat F108, L92 and binary mixed 0.5 wt % L92 + 1 wt % F108 system. Table S2. Hydrodynamic Diameters of neat F98, L92 and binary mixed 0.5 wt % L92 + 1 wt % F98 system. Table S3. Hydrodynamic Diameters of neat F88, L92 and binary mixed 0.5 wt % L92 + 1 wt % F88 system. Table S4. Hydrodynamic Diameters of neat F68, L92 and binary mixed 0.5 wt % L92 + 1 wt % F68 system. Table S5. Hydrodynamic Diameters of neat F87, L92 and binary mixed 0.5 wt % L92 + 1 wt % F87 system. Table S6. Hydrodynamic Diameters of neat P84, L92 and binary mixed 0.5 wt % L92 + 1 wt % F84 system.
