# Erratum: Synergistic Effect of Binary Mixed-Pluronic Systems on Temperature Dependent Self-Assembly Process and Drug Solubility. *Polymers* 2018, *10*, 105.

**DOI:** 10.3390/polym10060593

**Published:** 2018-05-28

**Authors:** Chin-Fen Lee, Hsueh-Wen Tseng, Pratap Bahadur, Li-Jen Chen

**Affiliations:** 1 Department of Chemical Engineering, National Taiwan University, Taipei 10617, Taiwan; nattianan24@gmail.com (C.-F.L.); r00524034@ntu.edu.tw (H.-W.T.); 2 Department of Chemistry, Veer Narmad South Gujarat University, Surat 395007, India; pbahadur2002@yahoo.com

The authors wish to make changes to the above-mentioned published paper [1]. The solubility data of ibuprofen were overestimated by 20%–36% due to improper reference cell calibration. All the solubility data of ibuprofen reported in Table 2 and Table 3 should be replaced by the following tables.

In addition, some statements related to these solubility data in the main body of the text should be modified. In abstract, the very last sentence should be replaced by: “The solubility of ibuprofen in the 0.5 wt % L92 + 0.368 wt % P84 system is as high as 2.71 mg/mL, which is 118 times higher than that in pure water at 37 °C”. In the conclusion section (page 15), the 5th sentence in the second paragraph should be changed to: “The capability to incorporate ibuprofen into the system L92 + P84 is most outstanding as it can incorporate up to 2.71 mg/mL, which is 118 times higher than that in pure water at 37 °C”.

Furthermore, some statements in Section 3.3 should be modified accordingly. However, the change does not affect the systematic tendencies observed from our experiments. The authors apologize for any inconvenience caused.

The change does not affect the scientific results. The manuscript will be updated and the original will remain online on the article webpage http://www.mdpi.com/2073-4360/10/1/105.

## Figures and Tables

**Table 2:** Hydrodynamic Diameter of aggregates (*D_h_*, nm) and solubility of ibuprofen in neat Pluronic Fx8 (1.5 wt %) and binary mixed Pluronic 0.5 wt % L92 + 1.0 wt % Fx8 systems at 37 °C.

Neat Pluronic	F108 (1.5 wt %)	F98 (1.5 wt %)	F88 (1.5 wt %)	F68 (1.5 wt %)
*D_h_* (nm) without ibuprofen	27.5 ± 0.4	25.5 ± 0.4	6.8 ± 0.2	5.6 ± 0.2
*D_h_* (nm) Saturated ibuprofen	26.6 ± 0.2	24.6 ± 0.2	23.9 ± 0.1	25.2 ± 1.8
Solubility of ibuprofen (mg/mL)	1.35 ± 0.03	1.35 ± 0.08	1.18 ± 0.01	0.44 ± 0.11
Mixed Pluronic	L92 (0.5 wt %) + F108 (1 wt %)	L92 (0.5 wt %) + F98 (1 wt %)	L92 (0.5 wt %) + F88 (1 wt %)	L92 (0.5 wt %) + F68 (1 wt %)
*D_h_* (nm) * without ibuprofen	30.3 ± 0.1	30.3 ± 1.5	632 ± 25	550 ± 85
*D_h_* (nm) Saturated ibuprofen	327 ± 20	314 ± 11	254 ± 8	590 ± 27
Solubility of ibuprofen (mg/mL)	2.28 ± 0.04	2.41 ± 0.07	2.25 ± 0.11	1.75 ± 0.07

* The data measured at 35 °C.

**Table 3:** Hydrodynamic Diameter of aggregates (*D_h_*, nm) and solubility of ibuprofen in neat Pluronic F8x and binary mixed Pluronic L92 + F8x systems at the same total mass concentration at 37 °C.

Neat Pluronic	F88 (1.5 wt %)	F87 (1.175 wt %)	P84 (0.868 wt %)
*D_h_* (nm) without ibuprofen	6.8 ± 0.2	5.7 ± 0.1	17.9 ± 0.8
*D_h_* (nm) Saturated ibuprofen	23.9 ± 0.1	20.1 ± 0.1	88.2 ± 0.04
Solubility of ibuprofen (mg/mL)	1.18 ± 0.01	1.51 ± 0.04	2.74 ± 0.03
Mixed Pluronic	L92 (0.5 wt %) + F88 (1 wt %)	L92 (0.5 wt %) + F87 (0.675 wt %)	L92 (0.5 wt %) + P84 (0.368 wt %)
*D_h_* (nm) * without ibuprofen	632 ± 25	406 ± 18	18.3 ± 1.0
*D_h_* (nm) Saturated ibuprofen	254 ± 8	144 ± 4	130 ± 7
Solubility of ibuprofen (mg/mL)	2.25 ± 0.11	2.28 ± 0.01	2.71 ± 0.24

* The data measured at 35 °C.
