# Supplementary material for: Synergistic Effect of Binary Mixed-Pluronic Systems on Temperature Dependent Self-assembly Process and Drug Solubility
Source: Polymers (Basel). 2018 Jan 22;10(1):105. doi: 10.3390/polym10010105 (PMC6415020; doi:10.3390/polym10010105)
Supplement: Supplementary file 1 [file polymers-10-00105-s001.pdf]

Supplementary Materials: Synergistic Effect of Binary Mixed-Pluronic Systems on Temperature Dependent Micellization Process and Drug Solubility

Chin-Fen Lee[1], Hsueh-Wen Tseng[1], Pratap Bahadur[2], Li-Jen Chen[1*]

1 Department of Chemical Engineering, National Taiwan University, Taipei 10617, Taiwan

2 Department of Chemistry, Veer Narmad South Gujarat University, Surat 395 007, India

* Correspondence: ljchen@ntu.edu.tw

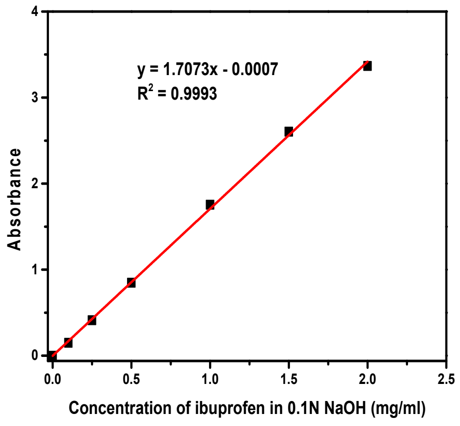

Figure S1. Calibration line of ibuprofen in 0.1 N NaOH solution.

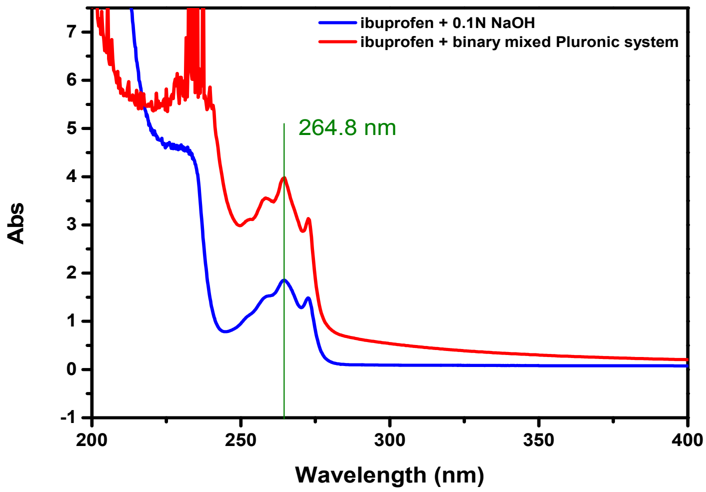

Figure S2. UV absorbance spectra of ibuprofen in different solvents. (blue line) in 0.1 N NaOH solution. (red line) in binary mixed Pluronic system.

There are some other studies in literature using UV-Vis technique to characterize physical-chemical properties of ibuprofen, such as the absorbance peak maximum λ_{max}. For example, Kesur et al. [1] found the λ_{max} = 265 nm for ibuprofen dissolved in 0.1N NaOH solution. Sunaric et al. [2] found the λ_{max} = 264 nm for ibuprofen dissolved methanol.

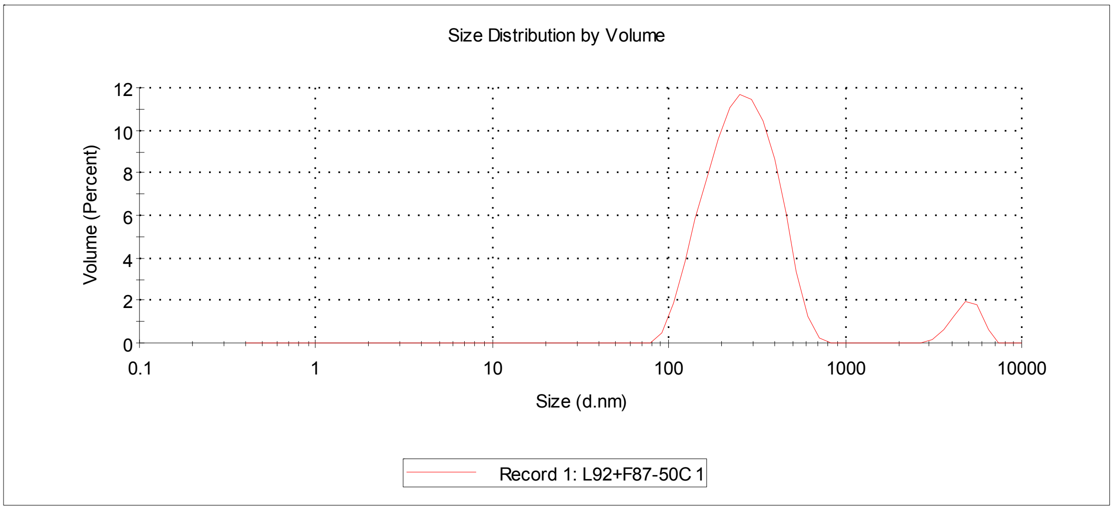

Figure S3. Volume based size distribution of system L92 + F87 at 50 °C

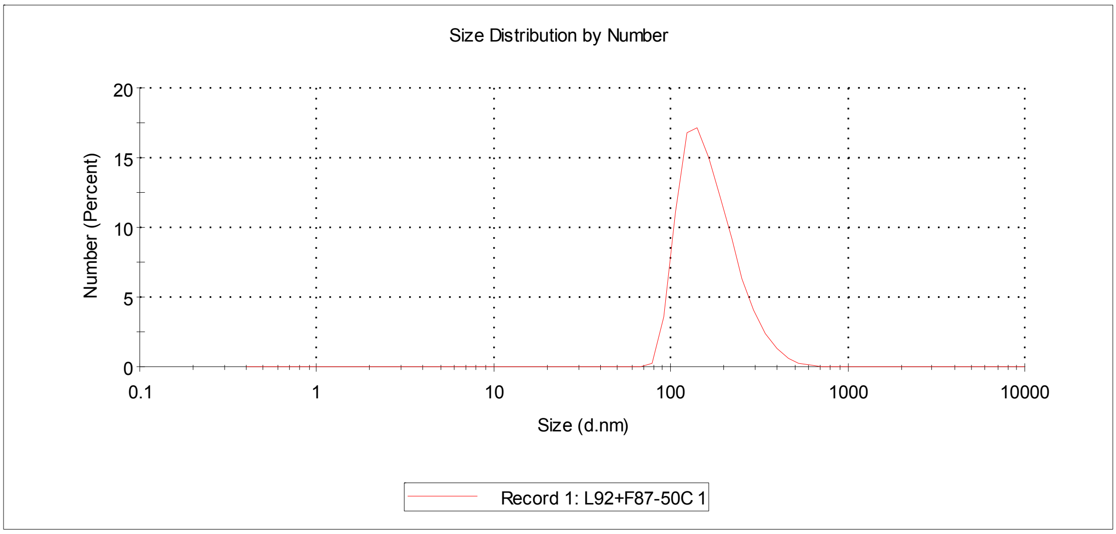

Figure S4. Number based size distribution of system L92 + F87 at 50 °C

Table S1. Hydrodynamic Diameters of neat F108, L92 and binary mixed system – 0.5wt% L92 + 1wt% F108.

**Systems**	**0.5wt% L92**		**1wt% F108**		**0.5 wt% L92 + 1 wt% F108**	
**Temperature (℃)**	**D (nm)**	**PDI**	**D (nm)**	**PDI**	**D (nm)**	**PDI**
15	3.86	0.99	8.05	0.24	6.63	0.49
20	135	0.12	8.71	0.27	188	0.26
25	262	0.20	8.25	0.26	191	0.14
30	476	0.15	8.82	0.39	37.9	0.46
35	450	0.15	26.1	0.15	30.3	0.11
40	456	0.19	24.1	0.06	28.9	0.09
45	450	0.20	23.8	0.04	28.5	0.07
50	449	0.20	24.0	0.03	28.4	0.07
55	404	0.11	23.9	0.02	28.7	0.08
60	311	0.06	23.8	0.01	28.8	0.08
65	285	0.02				
70	296	0.01				
75	321	0.01				
80	323	0.01				

Table S2. Hydrodynamic Diameters of neat F98, L92 and binary mixed system – 0.5wt% L92 + 1wt% F98.

**Systems**	**0.5wt% L92**		**1wt% F98**		**0.5 wt% L92 + 1 wt% F98**	
**Temperature (℃)**	**D (nm)**	**PDI**	**D (nm)**	**PDI**	**D (nm)**	**PDI**
15	3.86	0.99	7.24	0.09	5.75	0.46
20	135	0.12	7.49	0.11	282	0.20
25	262	0.20	7.76	0.16	358	0.23
30	476	0.15	8.27	0.23	36.3	0.78
35	450	0.15	5.73	0.26	30.3	0.57
40	456	0.19	26.2	0.14	28.7	0.54
45	450	0.20	24.6	0.13	28.8	0.53
50	449	0.20	24.9	0.12	27.9	0.52
55	404	0.11	24.9	0.10	27.6	0.55
60	311	0.06	24.5	0.16	27.8	0.61
65	285	0.02				
70	296	0.01				
75	321	0.01				
80	323	0.01				

Table S3. Hydrodynamic Diameters of neat F88, L92 and binary mixed system – 0.5wt% L92 + 1wt% F88.

Systems	0.5wt% L92		1wt% F88		0.5 wt% L92 + 1 wt% F88	
Temperature (℃)	D (nm)	PDI	D (nm)	PDI	D (nm)	PDI
15	3.86	0.99	6.96	0.17	5.57	1.00
20	135	0.12	7.20	0.23	170	0.19
25	262	0.20	7.59	0.25	284	0.16
30	476	0.15	7.70	0.28	464	0.28
35	450	0.15	7.69	0.25	633	0.44
40	456	0.19	22.9	0.26	381	0.45
45	450	0.20	21.9	0.12	29.7 (327)	0.49
50	449	0.20	20.4	0.06	26.6 (341)	0.52
55	404	0.11	20.4	0.04	25.1 (275)	0.48
60	311	0.06	20.5	0.04	25.8 (255)	0.47
65	285	0.02				
70	296	0.01				
75	321	0.01				
80	323	0.01				

Table S4. Hydrodynamic Diameters of neat F68, L92 and binary mixed system – 0.5wt% L92 + 1wt% F68.

Systems	0.5wt% L92		1wt% F68		0.5 wt% L92 + 1 wt% F68	
Temperature (℃)	D (nm)	PDI	D (nm)	PDI	D (nm)	PDI
15	3.86	0.99	6.22	0.23	4.83	1.00
20	135	0.12			137	0.51
25	262	0.20	6.31	0.22	263	0.26
30	476	0.15			727	0.38
35	450	0.15	6.16	0.23	551	0.34
40	456	0.19			578 (86.7)	0.31
45	450	0.20	6.50	0.25	415 (75.9)	0.23
50	449	0.20	7.57	0.22	350	0.21
55	404	0.11	17.8	0.20	467 (55.7)	0.34
60	311	0.06	17.1	0.12	559	0.32
65	285	0.02	18.7	0.16	638	0.41
70	296	0.01	16.7	0.05	524	0.35
75	321	0.01	17.5	0.04	381	0.29
80	323	0.01	17.0	0.04	316	0.26

Table S5. Hydrodynamic Diameters of neat F87, L92 and binary mixed system – 0.5wt% L92 + 0.675wt% F87.

Systems	0.5wt% L92		0.675wt% F87		0.5 wt% L92 + 0.675 wt% F87	
Temperature (℃)	D (nm)	PDI	D (nm)	PDI	D (nm)	PDI
15	3.86	0.99	7.28	0.24	4.97	0.52
20	135	0.12	6.64	0.24	142	0.19
25	262	0.20	6.51	0.23	243	0.13
30	476	0.15	6.93	0.27	391	0.24
35	450	0.15	6.36	0.23	406	0.27
40	456	0.19	19.4	0.24	294	0.31
45	450	0.20	18.0	0.11	250	0.34
50	449	0.20	16.6	0.02	245	0.37
55	404	0.11	16.7	0.03	243	0.35
60	311	0.06	16.8	0.03	225	0.36
65	285	0.02				
70	296	0.01				
75	321	0.01				
80	323	0.01				

Table S6. Hydrodynamic Diameters of neat P84, L92 and binary mixed system – 0.5wt% L92 + 0.368wt% P84.

Systems	0.5wt% L92		0.368wt% P84		0.5 wt% L92 + 0.368 wt% P84	
Temperature (℃)	D (nm)	PDI	D (nm)	PDI	D (nm)	PDI
15	3.86	0.99	4.90	0.44	3.81	0.48
20	135	0.12	5.40	0.35	86.1	0.46
25	262	0.20	5.24	0.36	246	0.22
30	476	0.15	4.77	0.40	492	0.34
35	450	0.15	16.5	0.13	18.3	0.69
40	456	0.19	15.4	0.06	19.2	0.55
45	450	0.20	15.1	0.03	29.4 (458)	0.53
50	449	0.20	16.0	0.06	46.4 (484)	0.63
55	404	0.11	16.8	0.06	54.1 (547)	0.62
60	311	0.06	47.4	0.16	52.2 (473)	0.59
65	285	0.02				
70	296	0.01				
75	321	0.01				
80	323	0.01				

References

1. Kesur, B.R.; Salunkhe, V.; Magdum, C. Development and validation of UV spectrophotometric method for simultaneous estimation of ibuprofen and famotidine in bulk and formulated tablet dosage form. *Int J Pharm Pharm Sci* **2012**, *4*, 271-274.

2. Sunaric, S.; Petkovic, M.; Denic, M.; Mitic, S.; Pavlovic, A. Determination of ibuprofen in combined dosage forms and cream by direct UV spectrophotometry after solid-phase extraction. *Acta Poloniae Pharm. Drug Res* **2013**, *70*, 401-411.
